# Associations between genetic risk, functional brain network organization and neuroticism

**DOI:** 10.1007/s11682-016-9626-2

**Published:** 2016-10-14

**Authors:** Michelle N. Servaas, Linda Geerligs, Jojanneke A. Bastiaansen, Remco J. Renken, Jan-Bernard C. Marsman, Ilja M. Nolte, Johan Ormel, André Aleman, Harriëtte Riese

**Affiliations:** 1 Neuroimaging Center, Department of Neuroscience, University of Groningen, University Medical Center Groningen, PO Box 196, 9700 AD Groningen, the Netherlands; 2 MRC Cognition and Brain Sciences Unit, University of Cambridge, 15 Chaucer Road, Cambridge, CB2 7EF UK; 3 Interdisciplinary Center for Psychopathology and Emotion regulation, Department of Psychiatry, University of Groningen, University Medical Center Groningen, PO Box 30.001, 9700 RB Groningen, the Netherlands; 4 Department of Epidemiology, University of Groningen, University Medical Center Groningen, PO Box 30.001, 9700 RB Groningen, the Netherlands; 5 Department of Psychology, University of Groningen, Grote Kruisstraat 2, 9712 TS Groningen, the Netherlands

**Keywords:** Functional connectivity, Genetics, Graph theory, Personality, Resting-state functional magnetic resonance imaging (rs-fMRI)

## Abstract

Neuroticism and genetic variation in the serotonin-transporter (*SLC6A4*) and catechol-O-methyltransferase (*COMT*) gene are risk factors for psychopathology. Alterations in the functional integration and segregation of neural circuits have recently been found in individuals scoring higher on neuroticism. The aim of the current study was to investigate how genetic risk factors impact functional network organization and whether genetic risk factors moderate the association between neuroticism and functional network organization. We applied graph theory analysis on resting-state fMRI data in a sample of 120 women selected based on their neuroticism score, and genotyped two polymorphisms: 5-HTTLPR (S-carriers and L-homozygotes) and COMT (rs4680-rs165599; COMT risk group and COMT non-risk group). For the 5-HTTLPR polymorphism, we found that subnetworks related to cognitive control show less connections with other subnetworks in S-carriers compared to L-homozygotes. The COMT polymorphism moderated the association between neuroticism and functional network organization. We found that neuroticism was associated with lower efficiency coefficients in visual and somatosensory-motor subnetworks in the COMT risk group compared to the COMT non-risk group. The findings of altered topology of specific subnetworks point to different cognitive-emotional processes that may be affected in relation to the genetic risk factors, concerning emotion regulation in S-carriers (5-HTTLPR) and emotional salience processing in COMT risk carriers.

**Electronic supplementary material:**

The online version of this article (doi:10.1007/s11682-016-9626-2) contains supplementary material, which is available to authorized users.

## Introduction

Neuroticism is a robust personality trait that is part of various widely accepted personality theories and models (a.o. Eysenck 1967), and can be defined as the tendency to react with a negative emotional response to life experiences (Ormel et al. 2013). It has been well established as a potent risk marker for a range of psychiatric disorders, particularly internalizing disorders (Lahey 2009). Furthermore, individuals scoring higher on neuroticism than average have more comorbid disorders, unexplained medical issues and general health problems (Lahey 2009). Consequently, this gives rise to considerable health care costs that even exceed those of common mental disorders (Cuijpers et al. 2010).

It is evident that neuroticism is a relevant concept for public health and that it is important to unravel its genetic basis and underlying neurobiological mechanisms. Prior research has indicated that neuroticism is moderately heritable, that is, approximately half of the variance can be explained by genetic factors (Riese et al. 2009). Two genes that have been associated with neuroticism and emotion processing are the serotonin transporter (*SLC6A4*) gene and catechol-O-methyltransferase (*COMT*) gene (for reviews, see Bevilacqua and Goldman 2011 and Canli 2008, though null-findings have also been found, see Genetics of Personality Consortium et al. 2015 and Terracciano et al. 2009). First, the *SLC6A4* gene is an important regulator of serotonergic neurotransmission and contains a prominent common length polymorphism 5-HTTLPR that encodes a short (S) and a long (L) allele (Bevilacqua and Goldman 2011). Carrying the S-allele has been associated with lower mRNA expression as well as lower serotonin uptake compared to carrying two copies of the L-allele (Lesch et al. 1996). However, in case of an A to G substitution in the single-nucleotide polymorphism (SNP) rs25531 located close to the 5-HTTLPR polymorphism, the transcriptional efficacy of the L-allele is rendered more comparable to the low-expressing S-allele (Hu et al. 2006; Wendland et al. 2006). The S-allele has been shown to explain inherited variance in neuroticism and other anxiety-related traits (Lesch et al. 1996).

Second, the *COMT* gene produces the enzyme COMT that inactivates catecholamine neurotransmitters (e.g. dopamine and norepinephrine) throughout the cerebrum (Hong et al. 1998), specifically the prefrontal cortex (PFC) (Egan et al. 2001). Enzyme function is in part influenced by a G to A substitution at codon 158 (rs4680), producing an amino acid change from valine (Val) to methione (Met) (Lachman et al. 1996). The Met-allele has a three-to-four fold reduction in enzyme activity compared to the Val-allele, leading to higher dopamine concentrations and more efficient information processing in the PFC (Egan et al. 2001; Lachman et al. 1996). A COMT haplotype containing the abovementioned SNP rs4680 and additional SNP rs165599 has been associated with neuroticism (risk haplotype: GG-AA) (Hettema et al. 2008), which potentially results in lower levels of cortical catecholamines (Bray et al. 2003). Notably, Hettema et al. (2008) found the Val-allele of SNP rs4680 to be the risk variant related neuroticism. This is in contrast with the warrior-worrier model, which states that individuals with the Val-allele of SNP rs4680 have an advantage in processing aversive stimuli, while individuals with the Met-allele have an advantage in tasks related to working memory (Stein et al. 2006). However, results from a recent meta-analysis (Lee and Prescott 2014) and review on meta-analysis studies (Gatt et al. 2015) conflict with the propositions of this model. For a discussion of possible explanations for this discrepancy, see aforementioned two articles. We chose to follow the results of the study of Hettema et al. (2008) because the authors investigated a shared genetic risk across a range of anxiety-related phenotypes and took other COMT functional loci into account, besides SNP rs4680.

As is the case with many complex mental disorders, the development of neuroticism is influenced by multiple genetic mutations of small effect (Canli 2008). For this reason, traditional association studies have regularly failed to find a link between such phenotypes and risk polymorphisms (Gottesman and Gould 2003). To overcome this problem, an approach was adopted that involves studying endophenotypes, which are intermediate phenotypes that lie in between the genotype (e.g. 5-HTTLPR) and phenotype (e.g. neuroticism) (Gottesman and Gould 2003). The assumption is that endophenotypes are more elementary in nature than phenotypes and because of that, implicate fewer genetic, environmental and epigenetic factors as well as interactions between them in producing phenotypic variation (Gottesman and Gould 2003). Since neuroticism has been related to alterations in brain functioning (Servaas et al. 2013), neural measures are ideal to be used as endophenotypes in the search for risk polymorphisms (i.e. imaging genetics) (Fornito et al. 2011; Fornito and Bullmore 2012). Recently, it has been proposed that psychopathology probably does not arise from dysfunctional activation in a few specific brain regions during a particular task, but from alterations in the functional integration and segregation of neural circuits (i.e. disrupted connectivity) (Fornito et al. 2011; Fornito and Bullmore 2012; Meyer-Lindenberg 2012). In line with this, we showed an altered functional network organization in individuals scoring higher on neuroticism than average (Servaas et al. 2015). Specifically, we found that the whole-brain network structure resembled more that of a random network and had overall weaker functional connections. Furthermore, we reported that neuroticism was associated with i) higher local efficiency coefficients (a measure used to define functional integration within a subnetwork) in the affective subnetwork (AS), ii) more connections (measured with the participation coefficient; a measure used to define functional integration between subnetworks) between the cingulum-operculum (salience) subnetwork (COS) and other functional subnetworks, and iii) lower local efficiency coefficients in sensory(−motor) (somatosensory-motor; SMS and visual; VS) and cognitive control (default mode; DMS and fronto-parietal; FPS) subnetworks.

As genetic data of the 120 individuals in the abovementioned study (Servaas et al. 2015) became available, we were interested to investigate whether genetic variation in the 5-HTTLPR and COMT polymorphism explains the reported associations between functional network organization and neuroticism. Furthermore, we were also interested to explore the main effect of genetic risk on functional network organization, since these genes have been related to multiple forms of psychopathology, not only neuroticism (Bevilacqua and Goldman 2011; Canli 2008). As a potential underlying biological mechanism, Hahn et al. (2011, 2013) speculated that risk polymorphisms may influence neural network plasticity (in interaction with the (early) environment) via neurotransmission during life (specifically development), leading to changes in behavior. We believe the connectomics method is a suitable choice in this endeavor because i) most genetic variants have distributed effects on brain functioning via, for example, alterations in neurotransmitter release or synaptic functioning (Fornito and Bullmore 2012), ii) there have been recent successes in applying this method to investigate the effect of other genetic variants (e.g. ε4 allele of the APOE gene*)* on (subnetwork) brain connectivity (Fornito and Bullmore 2012), iii) connectomic measures, compared to measures based on activations or single connections, have a higher signal-to-noise ratio and are more stable due to the elimination of weaker connections via proportional thresholding (Thompson et al. 2013) and iv) the polymorphisms COMT and 5-HTTLPR are two of the thirteen common variants found in a review of meta-analysis studies of candidate genes that are common in two or more psychiatric disorders (Gatt et al. 2015, see Fig. 2 of this article; notably, these genetic variants did not show overlap with variants identified in 12 meta-analyses of genome-wide association studies for the same disorders), which seems to indicate that these polymorphisms affect one or more functions that have a large impact on behavior/mental processing. In the current study, we hypothesized to find a whole-brain functional network organization that holds less small-world characteristics in genetic risk carriers compared to non-risk carriers (see method section for the group definition). This implies an imbalance between i) integration among brain regions (measured with the global efficiency coefficient) and ii) segregation of brain regions in specialized functional subnetworks (measured with the local efficiency and modularity coefficient). High integration and segregation are determined to be essential for optimal performance of complex systems such as the brain (Latora and Marchiori 2001). With regard to subnetwork topology, we hypothesized that subnetworks related to emotion processing have higher local efficiency coefficients and more connections with other subnetworks, than subnetworks related to cognitive control, in genetic risk carriers compared to non-risk carriers (Bevilacqua and Goldman 2011; Canli 2008).

## Methods

### Participants

This study was part of a larger project on the neural correlates of neuroticism (see Servaas et al. 2015 for further details). In short, 120 individuals (mean age: 20.8 SD ± 2.0, age range: 18–25) were selected from a larger sample of 240 students from the University of Groningen on the basis of their scores on the NEO Five-Factor Inventory (NEO-FFI) (domains Neuroticism and Extraversion, 24 items). To ensure sufficient numbers of participants with high levels of neuroticism, sixty individuals were selected from the highest quartile of neuroticism scores (NEO-FFI score ≥ 32, range 32–47) and sixty individuals were randomly selected from the three lowest quartiles (NEO-FFI < 32, range 17–31). Participants met the following selection criteria: 1) female gender, 2) age between 18 and 25 years, 3) Dutch as native language, 4) Caucasian descent, 5) right handed, 6) no use of contraceptive medication, except for oral contraceptive pills (21-pill packet). Only females were included because they tend to score higher on neuroticism and have a higher risk of developing affective disorders (Parker and Brotchie 2010). Furthermore, research is still limited related to gender differences in neuroticism. Therefore, we decided not to introduce this variation in the sample as it is not properly understood yet. Exclusion criteria were 1) a history of seizure or head injury, 2) a life time diagnosis of psychiatric and/or neurological disorders, 3) a life time diagnosis of psychiatric disorders in first degree relatives of the participant, 4) the use of medication that can influence test results, 5) visual or auditory problems that cannot be corrected, 6) MRI incompatible implants or tattoos, 7) claustrophobia, 8) suspected or confirmed pregnancy. All participants were scanned in the first ten days of their menstrual cycle or during the discontinuation week in case of oral contraceptive usage to control for menstrual cycle-related effects on neural correlates of mood, stress sensitivity and neurocognitive function (Andreano and Cahill 2010). On the day of the experiment, after explaining the procedure, participants gave informed consent and completed the NEO-PI-R (domains Neuroticism, Extraversion and Conscientiousness, 144 items) (Hoekstra et al. 1996). Plots of normality (QQ-plot and boxplot) showed that, in the selected 120 participants, neuroticism scores were approximately normally distributed. In addition, the study was approved by the Medical Ethical Committee of the University Medical Center Groningen and was conducted in accordance with the Declaration of Helsinki.

### Genotyping

DNA extraction and genotyping were performed at the Department of Laboratory Medicine of the University Medical Center Groningen, Groningen, the Netherlands. Saliva was collected in Oragene saliva collection and preservation kits (DNAGenotek, Ontario, Canada), and DNA was extracted according to the protocol of the manufacturer. For the *SLC6A4,* the 5-HTTLPR S/La/Lg variants were determined using a validated in-house method (Doornbos et al. 2009). In the remainder of this paper, we will use the term S allele for the Lg and S variants, and the term L allele for the La variant (Wendland et al. 2006). Genotyping of the COMT rs4680 and rs165599 SNPs was performed following the protocol supplied by Applied Biosystems (see Online resource 1, Supplement 1 for more details on the genotyping).

### Genotype analysis

We used PHASE (v2.1.1) (Stephens et al. 2001) to reconstruct two-marker haplotypes (a combination of alleles that are part of genotypes, which are likely to be inherited together) from COMT SNPs rs4680 and rs165599. Haplotype frequencies were determined and used to estimate the genotype probabilities of haplotype pairs. When a genotype probability exceeded 0.80, the corresponding haplotype pair was assigned. However, when all genotype probabilities were smaller than 0.80, haplotypes were set to missing. In the current study, all genotype probabilities exceeded 0.80. In the remainder of this paper, the haplotype COMT rs4680-rs165599 will be referred to as COMT.

Subjects were grouped into genetic risk carriers and non-risk carriers for the two polymorphisms (Hettema et al. 2008; Hu et al. 2006; Wendland et al. 2006). For the 5-HTTLPR polymorphism, the risk group includes the genotypes S/S and S/L, and the non-risk group includes the genotype L/L (Wendland et al. 2006); in the remainder of this paper denoted as S-carriers and L-homozygotes, respectively. For the COMT polymorphism, the risk group includes the genotypes Val/Val for rs4680 and/or A/A for rs165599, and the non-risk group includes the genotypes Met/Met or Val/Met for rs4680 and G/G or A/G for rs165599 (Hettema et al. 2008). In the remainder of this paper, we named the risk group “COMT risk” and the non-risk group “COMT non-risk”. The latter names were chosen for simplicity, since the groups consist of multiple SNP combinations, that is, the COMT risk group consists of individuals who are homozygote for either SNP (rs4680: Val/Val or rs165599: A/A) or both, and the COMT non-risk group consists of individuals who carry one of the other possible SNP combinations.

Hardy-Weinberg equilibrium was tested for the 5-HTTLPR, COMT rs4680, and COMT rs165599 polymorphism using a chi-square test with one degree of freedom.

### Image acquisition

A 3 Tesla Philips Intera MRI scanner (Philips Medical Systems, Best, the Netherlands), equipped with a 32-channel SENSE head coil, was used to acquire the images. A high-resolution T1-weighted 3D structural image was obtained using fast-field echo (FFE) for anatomical reference (170 slices; TR: 9 ms; TE: 8 ms; FOV: 256 × 231; 256 × 256 matrix; voxel size: 1 × 1 × 1 mm). Resting-state functional magnetic resonance imaging (rs-fMRI) images were acquired with a T2*-weighted gradient echo planar imaging (EPI) sequence. Participants were instructed to close their eyes and to not fall asleep. The scan comprised 300 volumes of 37 axial-slices (TR: 2000 ms; TE: 30 ms; FOV: 220 × 221; 64 × 62 matrix; voxel size: 3.5 × 3.5 × 3.5 mm). Slices were acquired in descending order without a gap. To prevent artifacts due to nasal cavities, images were tilted 10° to the AC-PC transverse plane (see Online resource 1, Supplement 2 for an overview of the full fMRI session).

### Data preprocessing

Image processing was performed using SPM8 (http://www.fil.ion.ucl.ac.uk/spm), implemented in Matlab 7.8.0 (Mathworks Inc., Natick, MA). Preprocessing included realignment, coregistration, DARTEL normalization (2x2x2 mm isotropic voxels) (Ashburner 2007) and smoothing (8 mm full-width at half maximum (FWHM) Gaussian kernel) (see Online resource 1, Supplement 3 for details on the preprocessing steps).

Next, a series of preprocessing steps specific to rs-fMRI analysis were performed. First, regression of several nuisance variables was applied per grey matter voxel to remove sources of spurious variance, comprising: six rigid body head motion parameters, the global signal, white matter (WM) signal and cerebrospinal fluid (CSF) signal. In order to obtain the last two signals, we performed segmentation of the T1-weighted image to create a WM and CSF mask and extracted the first eigenvariate from the time series of the included voxels. In addition, the first temporal derivatives of abovementioned nuisance variables were removed. Second, temporal band-pass filtering was applied to detrend the signal and to retain frequencies between 0.008–0.08 Hz (Van Dijk et al. 2010). Third, we performed scrubbing to additionally remove influences of movement on the rs-fMRI data (Power et al. 2012) (see Online resource 1, Supplement 4 for details on the scrubbing procedure).

After data preprocessing, nine participants were excluded from further analysis; two because of anatomical abnormalities (i.e. large ventricles that were still within the normal range but difficult to normalize), five because of technical difficulties, and two because of excessive scrubbing (viz. more than one-third of the volumes had to be removed). Furthermore, for the COMT polymorphism, two additional subjects had to be excluded due to failure of genotyping. The following total samples remained for statistical analysis: for the 5-HTTLPR polymorphism, a sample of 111 participants, and for the COMT polymorphism, a sample of 109 participants.

### Network construction

Network construction was previously applied in Servaas et al. (2015). Nodes (i.e. brain regions of interest) were built by creating a sphere of 5 mm radius around 264 coordinates provided by Power et al. (2011). After visual inspection of the regions of interest (ROIs), we noted the absence of three relevant subcortical structures for research on neuroticism: bilateral amygdala, hippocampus and caudate (Servaas et al. 2013). The coordinates for these regions were determined using the Harvard-Oxford Subcortical Structural Atlas. First, we thresholded the Harvard-Oxford atlas files at probability 80 % and second, we selected coordinates with the highest probability for the left and right amygdala, hippocampus and caudate separately. This resulted in a total of 270 ROIs. No overlap was observed between the additional ROIs and the ROIs of Power et al. (2011). Next, a whole-brain group mask was built based on the EPI images to locate the parts of the brain that are free from susceptibility artifacts in all subjects. Subsequently, we checked whether nodes overlapped more than 50 % (voxel-wise) with the group mask. This resulted in the exclusion of eleven ROIs. To construct a connectivity matrix per subject, we extracted the regional mean time series for each of the remaining 259 ROIs and calculated Pearson correlations between all pairs. Furthermore, to prevent biases due to shared non-biological signal between adjacent ROIs, correlations were set to zero when the distance was less than 20 mm between the centres of two ROIs (Power et al. 2011) (540 connections, 1.62 %). In addition, correlations on the diagonal of the connectivity matrix were set to zero as well.

### Thresholding and module index

**Fig. 1:**
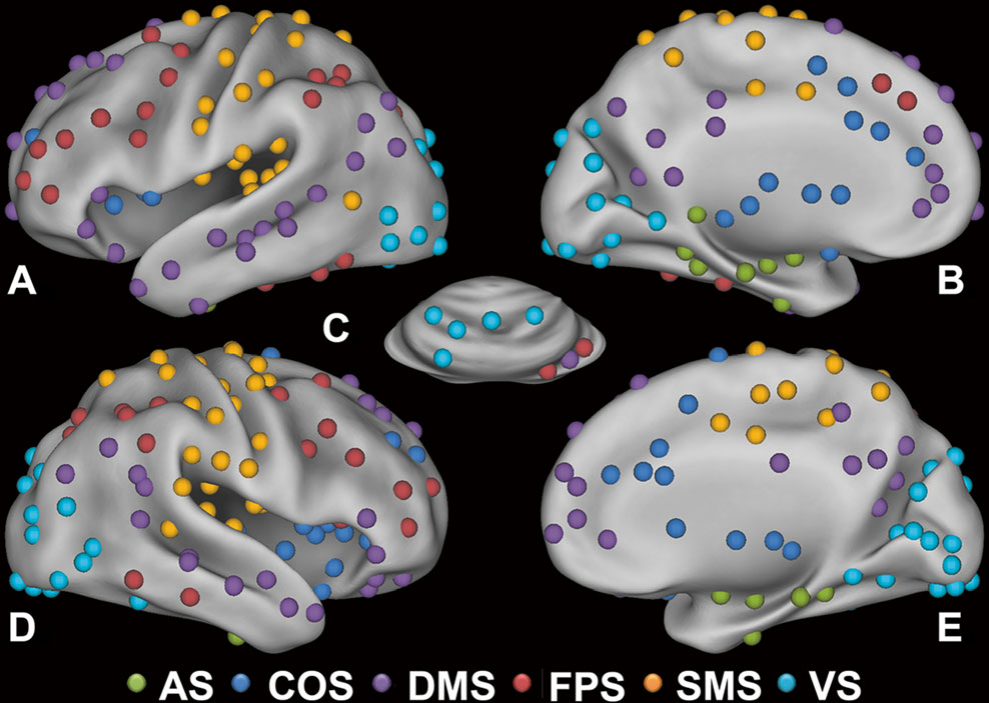
Module decomposition. Nodes could be partitioned in six functional subnetworks with a maximum number of within-group edges and a minimum number of between-group edges. Colors indicate the different modules that nodes belong to: AS, affective subnetwork (green); COS, cingulo-operculum subnetwork (dark blue); DMS, default mode subnetwork (purple); FPS, fronto-parietal subnetwork (red); SMS, somatosensory-motor subnetwork (orange); VS, visual subnetwork (light blue). Nodes are pasted on an inflated surface rendering of the human brain using the program CARET (v5.65). In the panels, different views are shown: A. left lateral, B. left medial, C. cerebellum dorsal, D. right lateral, E. right medial (reprinted from Servaas et al. 2015)

We applied a range of proportional thresholds to each correlation matrix per subject to avoid the confound of discrepant results on network measures, due to their sensitivity to the number of edges (i.e. connections) in a graph (van Wijk et al. 2010). The threshold values ranged from 1 % to 30 % in increments of 1 %. Network measures were calculated across the selected range of threshold values. Subnetworks were derived from the whole-brain graph by applying the algorithm of Blondel et al. (2008) and the modularity fine-tuning algorithm of Sun et al. (2009) (see Online resource 1, Supplement 5 for details on the module decomposition). For this procedure, we selected a single optimal threshold by using the method of Geerligs et al. (2015). The optimal threshold in the current study was 1.8 % (see Online resource 1, Supplement 6 for details on the selection of the optimal threshold). In total, six subnetworks could be derived with a maximum number of within-group edges and a minimum number of between-group edges (Rubinov and Sporns 2010). These included the AS, COS, DMS, FPS, SMS and VS (see Fig. 1).

### Network measures

Network measures were calculated on weighted (i.e. edge strengths are preserved) graphs across the selected range of threshold values by using functions implemented in the Brain Connectivity Toolbox (www.brain-connectivity-toolbox.net, Rubinov and Sporns 2010). First, we calculated the whole-brain network measures: global efficiency, local efficiency (averaged across nodes) and maximized modularity. Global efficiency is calculated as the average inverse shortest path length between all pairs of nodes. Local efficiency is calculated in a similar manner, but then between the neighbours of a specific node (Latora and Marchiori 2001; Rubinov and Sporns 2010). Maximized modularity is calculated with a function that quantifies the degree to which a network can be clearly delineated in non-overlapping groups of nodes (Rubinov and Sporns 2010). Second, we calculated local efficiency (averaged across nodes) and the participation coefficient (averaged across nodes) per module. The participation coefficient is calculated as the ratio of intra- versus intermodular connections per node (Rubinov and Sporns 2010). For the interaction analyses, we only examined network measures that were related to neuroticism in our previous paper (Servaas et al. 2015) to investigate whether genetic variation in the 5-HTTLPR and COMT polymorphism explains the reported associations between functional network organization and neuroticism. These include all three whole-brain network measures, local efficiency for the modules DMS, FPS, SMS and VS, and the participation coefficient for the modules COS and SMS.

### Network analyses

Across the selected range of threshold values, we calculated i) the mean difference between the genetic risk and non-risk group per network measure for both polymorphisms, ii) the difference in slope between the genetic risk and non-risk group for the association between neuroticism and a specific network measure for both polymorphisms. These difference measures were plotted and visually checked for consistency across threshold values. Subsequently, we calculated the AUC across threshold values per network measure for both polymorphisms to obtain a summarized scalar that is independent of single threshold selection. Next, non-parametric permutation testing was applied on the AUC per network measure to assess whether the results could have occurred by chance. To this end, genetic group membership was permuted randomly and the difference measures were recalculated. This procedure was repeated 5000 times and a two-tailed test of the null hypothesis (*p* < 0.05) was performed (Zhang et al. 2011).

## Results

### Sample characteristics

The mean NEO Personality Inventory Revised (NEO-PI-R) neuroticism score across the whole sample was 135.5 SD ± 18.9 (range: 94–195). The genotype and allele frequencies closely resembled findings from the European HapMap31 (5-HTTLPR, L/L = 31, L/S = 57, S/S = 32, L = 0.50, S = 0.50; COMT rs4680, A/A = 34, A/G = 57, G/G = 28, A = 0.53, G = 0.47; COMT rs165599, A/A = 56, A/G = 51, G/G = 11, A = 0.69, G = 0.31) (Hu et al. 2006; Wendland et al. 2006). Genotype distributions were in Hardy-Weinberg equilibrium (5-HTTLPR, *p* = 0.58; COMT rs4680, *p* = 0.67; COMT rs165599, *p* = 0.90). For the 5-HTTLPR polymorphism, the S-carrier group comprised 80 individuals and the L-homozygote group comprised 31 individuals. For the COMT polymorphism, the COMT risk group comprised 42 individuals and the COMT non-risk group comprised 67 individuals. For both polymorphisms, the genetic risk group did not significantly differ from the genetic non-risk group based on their mean neuroticism scores (5-HTTLPR t_(109)_ = −0.72, *p* = 0.48; COMT t_(107)_ = −0.07, *p* = 0.95). For a table with the mean neuroticism scores per genetic group, see Online resource 1, Supplement 7, Table 1.

### Network measures

#### Main effect of genetic group

**Fig. 2:**
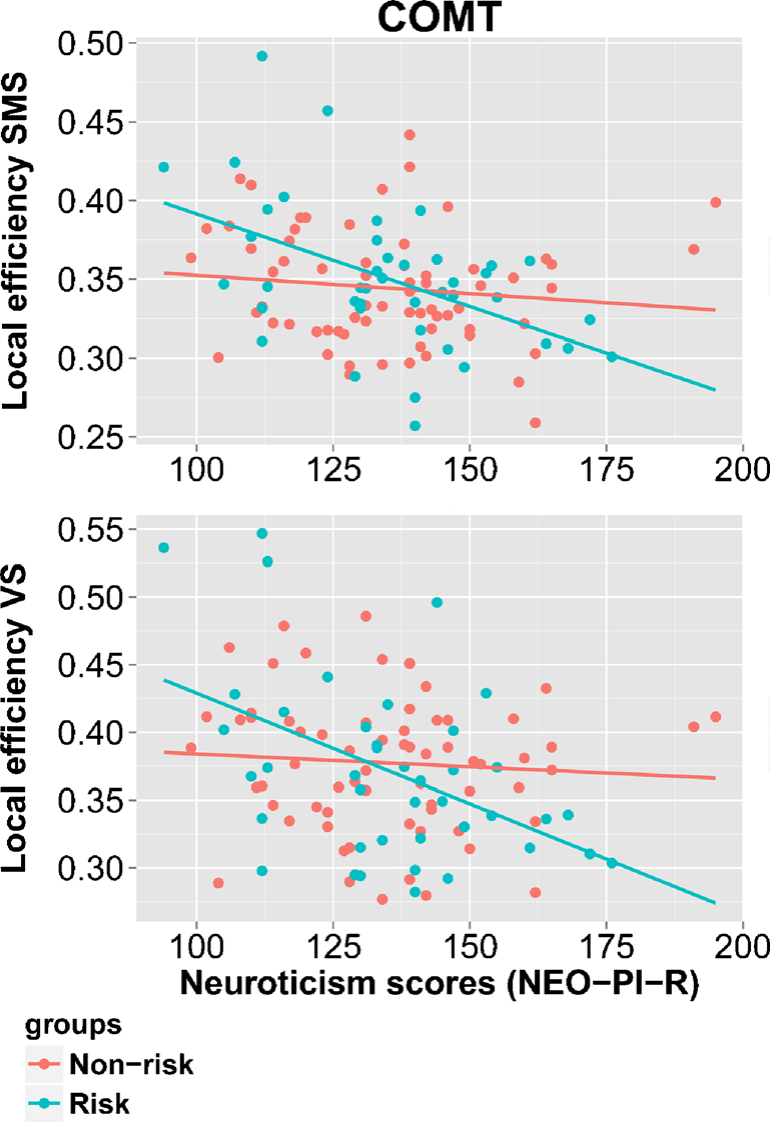
Results for the interaction between the COMT polymorphism and neuroticism. In the SMS and VS, neuroticism was negatively correlated with local efficiency in the risk COMT group, while a weak correlation was observed in the non-risk COMT group. Results are visualized for the proportional threshold of 15 %. The figure was created with the package ggplot2 in R (v0.98.1062). COMT, catechol-O-methyltransferase, NEO-PI-R, NEO personality inventory revised; SMS, somatosensory-motor subnetwork; VS, visual subnetwork

For the 5-HTTLPR polymorphism, we found a decreased participation coefficient in the DMS (*p* = 0.033) and FPS (*p* = 0.015) in S-carriers compared with L-homozygotes. No significant results were identified for whole-brain network measures or the COMT polymorphism (see Online resource 1, Supplement 8, Table 2 for all statistic results and Supplement 9, Fig. 1 and Supplement 10, Fig. 2 for density plots and boxplots of the results to gain more insight in the differences between S-carriers and L-homozygotes for the participation coefficient of the DMS and FPS).

#### Interaction effect between genetic group and neuroticism

The COMT polymorphism moderated the association between neuroticism and local efficiency in the SMS (*p* = 0.050) and VS (*p* = 0.023). In these two subnetworks, neuroticism was negatively correlated with local efficiency in the COMT risk group, while a weak correlation was observed in the COMT non-risk group (see Fig. 2 for scatter plots of the results and see Online resource 1, Supplement 11, Table 3 for the specific correlation values per group for each threshold value). No significant results were identified for whole-brain network measures or the 5-HTTLPR polymorphism (see Online resource 1, Supplement 8, Table 1 for all statistic results and see Supplement 12, Fig. 3 and Supplement 13, Fig. 4 for the bootstrap results to gain more insight in the stability of the correlation slopes for the association between neuroticism and local efficiency in the SMS and VS per COMT group).

A posthoc check was performed by reanalyzing all statistical tests using binary (i.e. an edge is present or not) graphs, instead of weighted graphs (i.e. edge strengths are preserved). Binary graphs provide information on the functional structure of the network organization, and weighted graphs additionally provide information on the functional connectivity strength. In the current study, we focused on the latter because it includes both types of information. The reason for the posthoc check was that we found discrepant results between both types of graph in our previous paper (Servaas et al. 2015) and we sought to verify whether this was the case for the current study. The results were comparable, except that we did not find a significant moderating effect of the COMT polymorphism on the association between neuroticism and local efficiency in the SMS for binary graphs (*p* = 0.138). Though, the effect was in a similar direction, that is, the negative correlation slope was stronger in the COMT risk group than the COMT non-risk group.

## Discussion

The aim of the current study was to investigate associations between genetic risk, functional network organization and neuroticism. Whereas previous work showed both whole-brain and subnetwork alterations related to neuroticism (Servaas et al. 2015), genetic risk factors were only associated with alterations on the subnetwork level. For the 5-HTTLPR polymorphism, we found less involvement of cognitive control subnetworks (DMS and FPS) in the functional network organization of S-carriers compared to L-homozygotes. Furthermore, the COMT polymorphism, not the 5-HTTLPR polymorphism, moderated the association between neuroticism and functional network organization. Individuals scoring higher on neuroticism than average, compared to individuals with lower scores, showed lower local efficiency coefficients for visual and somatosensory-motor subnetworks in the COMT risk group compared to the COMT non-risk group. No main effects were identified for the COMT polymorphism on functional network organization.

For the 5-HTTLPR polymorphism, we observed that the DMS and FPS had relatively fewer connections with other functional subnetworks in S-carriers compared to L-homozygotes. In contrast to L-carriers, S-carriers (i.e. risk allele) have been reported to show an attentional bias (Beevers et al. 2009; Pérez-Edgar et al. 2010) and heightened emotional reactivity (Hariri et al. 2002; Heinz et al. 2005; Pezawas et al. 2005) towards negative emotional stimuli.^1^ A potential underlying mechanism may be less efficient cognitive control of PFC regions over limbic structures (for a recent review, see Jonassen and Landro 2014). In line with this proposition, studies have found both increased activation in PFC regions and reduced functional connectivity between the amygdala and PFC regions during emotion processing and executive functioning in S-carriers compared to L-carriers (Jonassen et al. 2012; Stollstorff et al. 2013; Surguladze et al. 2008; Volman et al. 2013). Furthermore, structural studies have shown reduced grey matter density in PFC regions (Canli et al. 2005) and reduced white matter integrity of the uncinate fasciculus, an amygdala-PFC tract (Pacheco et al. 2009), in S-carriers. In addition, during reappraisal of negative emotional pictures, individuals homozygous for the S-allele showed no reductions in negative mood and increased activation of the superior frontal gyrus and anterior insula, compared to individuals homozygous for the L-allele (Firk et al. 2013). These findings may indicate that S-carriers show less efficient and less effective top-down cognitive control over negative emotions compared to L-carriers. In accordance, we found fewer connections between cognitive control subnetworks and other functional subnetworks in S-carriers compared to L-homozygotes in the current study. Notably, we did not find evidence for alterations in subnetworks related to emotion processing. It seems possible that, specifically, cognitive control over these subnetworks is impaired but not functioning of the emotion subnetworks themselves. This is in line with our previous work, wherein we casted doubt on the association between the 5-HTTLPR polymorphism and amygdala activation (Bastiaansen et al. 2014). Furthermore, we did not find a moderating effect of the 5-HTTLPR polymorphism on the association between functional network organization and neuroticism. This may indicate that the association between the 5-HTTLPR polymorphism, brain functioning and neuroticism is dependent on other factors, such as epistasis, pleiotropy or gene-by-environment interactions (Canli 2008; Hahn et al. 2011, 2013). In conclusion, our findings may suggest that S-carriers are more sensitive and reactive to negative emotional stimuli, due to hampered top-down emotion regulation.

For the COMT polymorphism, we found that neuroticism was associated with lower local efficiency coefficients for the VS and SMS in the COMT risk group compared to the COMT non-risk group. In our previous study of this sample (Servaas et al. 2015), we also found a negative correlation between neuroticism and local efficiency for the VS and SMS. The current results indicate that this is specifically the case in COMT risk carriers. Previous research has shown that noradrenaline, one of the catecholamines that is degraded by the enzyme COMT, modulates sensory processing in auditory, visual and somatosensory pathways (Coull 1998; Sara 2009). This neuromodulator fine-tunes neural responses and improves signal-to-noise ratio, gating and spike synchrony in response to sensory stimuli to facilitate perceptual acuity (Sara 2009). Furthermore, it favours novelty and plays an important role in perceptual rivalry to effectively adapt to salient environmental events (Coull 1998; Sara 2009; Schultz and Dickinson 2000). In line with this, the COMT polymorphism, in interaction with the dopamine transporter (DAT1) polymorphism, has been related to worse task performance and alterations in amplitudes of event-related potential (ERP) components during visual and motor post-processing in a continuous performance task (Bender et al. 2012a, b). In addition, a recent connectomics study found decreased eigenvector centrality (a measure that quantifies the relative importance of a node) values in brain areas part of the somatomotor network in Val/Val carriers (rs4680 i.e. risk carriers) compared to Met carriers (Markett et al. 2015). Accordingly, we found a negative association between neuroticism and local efficiency for sensory subnetworks in the COMT risk group compared to the COMT non-risk group. Aforementioned findings may indicate that COMT risk carriers scoring higher on neuroticism than average show less efficient and/or effective sensory processing, specifically during situations of biological significance. This relates to the hypothesis of impaired associative learning in high neurotic individuals, leading to difficulties in predicting and adaptive responding to salient (emotional) stimuli (Servaas et al. 2013). However, we did not find a main effect of the COMT polymorphism on functional network organization. It may be possible that COMT risk carriers are able to functionally compensate for the differences in sensory processing, but that this is not the case for individuals who also score higher on neuroticism than average. In conclusion, our findings may suggest that COMT risk carriers, scoring higher on neuroticism than average, are more sensitive to stress and negative emotions, due to impaired processing of salient (emotional) stimuli in their environment.

It is interesting to note that the effects of neuroticism on functional network organization, which we found in our previous study (Servaas et al. 2015), are more pronounced than the effects of polymorphisms on functional network organization. We only found genetic effects on the subnetwork level, not the whole-brain level. Possibly, genetic effects are more specific or they will become more evident, when the joint effect of multiple polymorphisms are analysed. Furthermore, we did not find an association between genetic risk and neuroticism. This is in line with former studies investigating associations between genetic risk, brain functioning and personality (Hahn et al. 2011, 2013), underlying the need for applying the endophenotype approach in genetic neuroimaging. In addition, the results of the current study should be considered exploratory, because of the number of statistical tests that were performed. The presented *p*-values for type I error correction may be inflated. We tried to alleviate the multiple comparison problem in several ways: i) we checked whether results were consistent across threshold values by calculating the area under the curve (AUC), ii) we reduced the number of tests by calculating a mean of the nodal network measures per subnetwork, iii) we limited the number of chosen network measures to three on the whole-brain level and to two on the subnetwork level, iv) for the interaction analyses, we only examined network measures that were related to neuroticism in our previous paper (Servaas et al. 2015) to investigate whether genetic variation in the 5-HTTLPR and COMT polymorphism explains the reported associations between functional network organization and neuroticism. Moreover, we i) calculated our results on binary as well as weighted graphs as a robustness check, ii) performed permutation testing on the AUC to assess whether results could have occurred by chance and iii) created density and boxplots and performed bootstrapping to gain insight in the size and stability of the found results, respectively. Notably, it is difficult to adequately correct for multiple comparisons in graph analyses, since network measures are not independent from each other (mean *r* = 0.48, threshold 15 %). Multivariate methods would be more ideal to apply and are currently being developed (Simpson et al. 2013). However, a downside of multivariate methods is that results may be less interpretable. Notwithstanding our efforts, we believe that our results are in definite need of replication. Until then, our results should be carefully interpreted. Our study can be seen as one of the contributions to the field, wherein we try to unravel genetic influences on brain functioning to learn more about the etiology of psychopathology. Future meta-analyses should reveal whether our findings are consistently found across other studies and whether the connectomic approach is indeed more fruitful than the previous approach wherein we investigated activations in and connections between a few specific brain regions.

Furthermore, several other limitations of this study need to be considered. First, we had no direct measures of serotonin and dopamine levels in the different functional subnetworks. Second, although our sample size is relatively large, it was too small to investigate interactions between the two polymorphisms 5-HTTLPR and COMT. Third, we only investigated female students, and therefore, our findings cannot be generalized to the whole population. Future studies should replicate our results in male and older samples, and samples with a lower social economic status or a different ethnicity. However, by selecting a homogenous sample, we controlled for several important confounders, such as gender, age, education level and ethnicity. Fourth, we tested associations between genetic risk, functional network organization and neuroticism. For future research, it would be of interest to determine causal relationships between these factors to investigate the validity of models proposed by the endophenotype approach (e.g. mediational model) (Kendler and Neale 2010). Does polymorphic-dependent neurotransmission indeed influence neural (network) plasticity (in interaction with the (early) environment) that causes impaired emotion regulation or salience processing (Hahn et al. 2011, 2013)? Though interesting, it is challenging to investigate, since i) functioning of neurotransmission is extremely complex, ii) causal effects are difficult to determine, iii) there are other unknown factors at work (e.g. pleiotropy, epistasis and gene-by-environment interactions) and iv) pathways have small effect sizes (Canli 2008; Hahn et al. 2011, 2013). More in vitro (e.g. gene expression quantification), in vivo (e.g. single photon emission computed tomography, SPECT) and longitudinal studies are necessary to unravel causal relationships between genetic risk, brain functioning and neuroticism. Fifth, since network measures were calculated on rs-fMRI data in the current study, our results should be replicated using tasks that explicitly investigate emotion regulation or salience processing.

## Conclusions

Our findings may suggest that i) S-carriers are more sensitive and reactive to negative emotional stimuli, because of hampered top-down emotion regulation and ii) COMT risk carriers, who score higher on neuroticism than average, are more sensitive to stress and negative emotions, due to impaired processing of salient (emotional) stimuli in their environment. These findings of altered topology of specific subnetworks may help explain why genetic risk carriers (scoring higher on neuroticism than average) show less adaptive emotion processing and are more prone to develop psychopathology.

## Electronic supplementary material

ESM 1 (PDF 1.01 mb)

## Footnotes

1 Note that studies, cited in the subsection on the 5-HTTLPR polymorphism of the discussion, used different genotype group definitions: i) S-homozygotes versus L-carriers (Volman et al. 2013), ii) S-carriers versus L-homozygotes (Beevers et al. 2009; Canli et al. 2005; Hariri et al. 2002; Pezawas et al. 2005), iii) S-homozygotes versus L-homozygotes (Beevers et al. 2009; Firk et al. 2013; Stollstorff et al. 2013), iv) S-homozygotes versus heterozygotes versus L-homozygotes (Jonassen et al. 2012; Pérez-Edgar et al. 2010; Surguladze et al. 2008). In sum, it varies per study whether the heterozygote group is included in the SS or LL group, is omitted or is treated as an independent group. For this reason, we used the terms S-carriers and L-carriers when describing the results of studies using different genotype group definitions.
