# Supplementary material for: Associations between genetic risk, functional brain network organization and neuroticism
Source: Brain Imaging Behav. 2016 Oct 14;11(6):1581–91. doi: 10.1007/s11682-016-9626-2 (PMC5707236; doi:10.1007/s11682-016-9626-2)
Supplement: Supplementary file 1 — (PDF 1.01 mb) [file 11682_2016_9626_MOESM1_ESM.pdf]

SUPPLEMENTARY MATERIAL

Title Associations between genetic risk, functional brain network organization and neuroticism

Journal name Brain Imaging and Behavior

Author names Michelle N. Servaas[1*], Linda Geerligs[2], Jojanneke A. Bastiaansen[3], Remco J. Renken[1], Jan-Bernard C. Marsman[1], Ilja M. Nolte[4], Johan Ormel[3], André Aleman[1,5]; Harriëtte Riese[3]

Corresponding author M.N. Servaas

Neuroimaging Center, Department of Neuroscience

University of Groningen, University Medical Center Groningen

E-mail: m.n.servaas@umcg.nl

Supplement 1: Genotyping

For the *SLC6A4*, the 5-HTTLPR S/La/Lg variants were determined using PCR with Forward primer FAM-5'TGAATGCCAGCACCTAACCC-3' and Reverse primer 5-TTCTGGTGCCACCTAGACGC-3' (35 cycli of 30 seconds at 95°C, 30 seconds at 61°C and 1 minute at 72°C), and subsequent ingestion of the PCR product with the restriction enzyme Msp-I for at least 3 hours at 37 °C. The resulting restriction fragments were separated using capillary electrophoresis (ABI 3130 analyzer; Applied Biosystems, Nieuwerkerk a/d IJssel, the Netherlands), and fragment lengths were estimated using the ABI Prism® GeneMapper™ software, version 3.0 (Applied Biosystems). The La, Lg and S variants were determined by the detection of fragments of 325 base pairs (bp), 152 bp and 284 bp, respectively (validated in-house method, (Doornbos et al. 2009)).

Genotyping of the COMT rs4680 polymorphism (1947 G/A; $Val_{158}Met$; GenBank Z26491) was performed with the allelic discrimination technique following the protocol supplied by Applied Biosystems. We used primers COMT-GAF (5'-CGAGATCAACCCCGACTGT-3') and COMT-GAR (5'-CAGGCATGCACACCTTGTC-3'), and minor grove-binding probes VIC-5'-TTTCGCTGGCGTGAAG-3'-NFQ (G) and FAM-5'-TCGCTGGCATGAAG-3'-NFQ (A). The COMT rs165599 polymorphism was genotyped using the commercially available kit C_2255335_10 (Applied Biosystems). All COMT reactions were carried out in TaqMan universal PCR master mix using a 7500 Real-Time PCR System (Applied Biosystems).

Supplement 2: Overview of the full fMRI session

The full fMRI session consisted of four tasks, resting state and an anatomical scan. The following tasks/scans were presented in consecutive order: emotional face matching task (Hariri et al. 2002), mood (worry) induction paradigm (Paulesu et al. 2010), anatomical scan, resting

state, interoceptive sensitivity task (Pollatos et al. 2007) and Ultimatum Game (Sanfey et al. 2003). The total duration of the fMRI session was approximately 60 minutes. The order was fixed and identical for all participants.

Supplement 3: Preprocessing steps

First, structural as well as functional images were reoriented parallel to the AC-PC plane. Second, functional images were realigned to the first image using rigid body transformations and the mean EPI image, created during this step, was coregistered to the anatomical T1 image. Third, structural images were corrected for bias field inhomogeneities, registered using linear transformations and segmented into grey matter (GM), white matter (WM) and cerebrospinal fluid (CSF) (MNI template space). Fourth, we used DARTEL (diffeomorphic anatomical registration through exponentiated lie algebra toolbox) (Ashburner 2007) to create a customized group template to increase the accuracy of inter-subject alignment. Individual GM and WM tissue segments were iteratively aligned to the group template in order to acquire individual deformation flow fields. Fifth, the coregistered functional images were normalized to MNI space using the customized group template and individual deformation flow fields. Furthermore, images were resampled to 2 mm^3 isotropic voxels and smoothed with an 8 mm full-width at half-maximum (FWHM) Gaussian kernel.

Supplement 4: Scrubbing procedure

The indices framewise displacement (FD) and DVARS were calculated to indicate volumes (i.e. frames) that may be affected by motion artifacts (Power et al. 2012). FD is calculated as the root of the sum of the squared differentials per volume. Rotations were converted to translations assuming a distance of 65 mm from the origin of rotation (ArtRepair toolbox, http://cibsr.stanford.edu/tools/human-brain-project/artrepair-software.html). DVARS is

calculated as the root mean square (RMS) of the derivatives of the time series across voxels included in the whole-brain mask per volume (Power et al. 2011, 2012). Volumes were removed when FD>0.5 mm and DVARS>mean + 3*SD. Additionally, one backward and two forward neighboring volumes were removed as well. The median of the number of scans that were removed per subject was 11.0 (IQR: 14.2). Subjects were excluded when more than one third of the volumes had to be removed. After scrubbing, neuroticism scores did not correlate with mean head displacement, maximum head displacement, head rotation and the number of micromovements (<0.1 mm) (p>0.17) (Van Dijk et al. 2010). Furthermore, neuroticism did not correlate with the number of removed scans (p=0.63).

Supplement 5: Module decomposition

A two-step procedure was applied to achieve the optimal modular structure using a threshold of 1.8% (see the next paragraph for details on the selection of this threshold). Input for this procedure was the binarized correlation matrix averaged across subjects. First, nodes were partitioned into modules using the algorithm of Blondel et al. (2008) (Blondel et al. 2008), wherein nodes are divided into groups with a maximum number of within-group edges and a minimum number of between-group edges. This calculation was repeated 500 times to increase the chance of escaping local maxima. The statistic was further optimized by applying the modularity fine-tuning algorithm of Sun et al. (2009) (Sun et al. 2009), wherein nodes are randomly assigned to other modules until modularity no further improves.

Supplement 6: Selection of the optimal threshold for module decomposition

First, correlation matrices were binarized using a range of threshold values (T=0.01-0.30, in increments of 0.01). Second, these matrices were averaged across subjects per threshold value and the entropy was calculated for each of them to indicate for which threshold value the edges

showed the largest stability information-wise (lowest entropy). These results were compared to results obtained via randomized matrices (for details, see (Geerligs et al. 2015)). The optimal threshold is the threshold where (i) the original matrix shows the largest stability across subjects (low entropy) and (ii) the difference in entropy is the largest between the original matrix and random matrix. The optimal threshold in the current study was 1.8%.

Supplement 7

Table 1: Mean neuroticism scores per genetic group

	Mean	SD
**5-HTTLPR**		
S-carrier group	136.18	19.67
L-homozygote group	133.26	18.15
**COMT**		
Risk group	135.38	19.05
Non-risk group	135.13	19.62

Table 1 Mean neuroticism scores per genetic group. COMT, catechol-*O*-methyltransferase; SD, standard deviation.

Supplement 8

Table 2: Statistic results on the network measures

Network measure	P-values AUC main effect	Direction	P-values AUC interaction	Direction
**5-HTTLPR**				
Whole-brain				
Global efficiency	0.128		0.264	
Local efficiency	0.067*	Risk>Non-risk	0.550	
Modularity	0.077*	Risk>Non-risk	0.967	
Subnetworks: local efficiency				
AS	0.589			
COS	0.227			
DMS	0.105		0.276	
FPS	0.180		0.830	
SMS	0.270		0.348	
VS	0.055*	Risk>Non-risk	0.817	
Subnetworks: participation coefficient				
AS	0.774			
COS	0.480		0.285	
DMS	0.034**	Risk<Non-risk		
FPS	0.015**	Risk<Non-risk		
SMS	0.309		0.201	
VS	0.098*	Risk<Non-risk		
**COMT**				
Whole-brain				
Global efficiency	0.953		0.117	
Local efficiency	0.879		0.061*	Risk>Non-risk
Modularity	0.224		0.802	
Subnetworks: local efficiency				
AS	0.529			
COS	0.361			
DMS	0.690		0.146	
FPS	0.820		0.423	
SMS	0.220		0.050**	Risk>Non-risk
VS	0.396		0.023**	Risk>Non-risk
Subnetworks: participation coefficient				
AS	0.378			
COS	0.633		0.472	
DMS	0.896			
FPS	0.162			
SMS	0.761		0.832	

VS	0.181

Table 2 Permutation results for the main effect of genetic group and the interaction between genetic group and neuroticism. For the main effect, the mean difference was calculated between the genetic risk and non-risk group per network measure for both polymorphisms. For the interaction effect, the difference in slope was calculated between the genetic risk and non-risk group for the association between neuroticism and a specific network measure for both polymorphisms. For the latter analyses, we only examined network measures that were related to neuroticism in our previous paper (Servaas et al. 2015). AS, affective subnetwork; COMT, catechol-*O*-methyltransferase; COS, cingulo-operculum subnetwork; DMS, default mode subnetwork; FPS, fronto-parietal subnetwork, SMS, somatosensory-motor subnetwork; VS, visual subnetwork. ** p-value ≤0.05, * p-value ≤00.10.

Supplement 9

Figure 1: Density plots and boxplots of the main effect of the 5-HTTLPR polymorphism (participation coefficient of DMS)

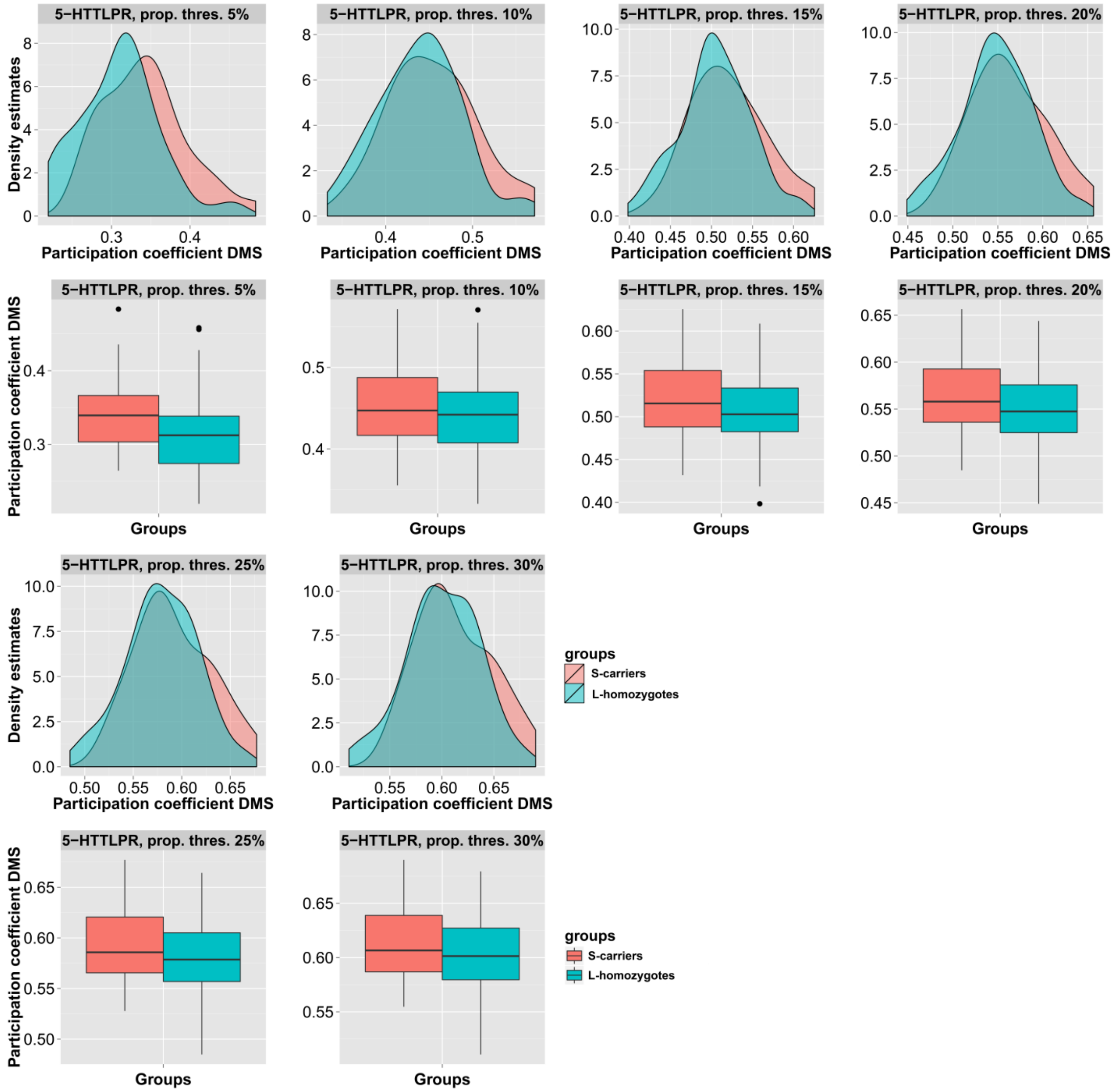

Figure 1 For the main effect of the 5-HTTLPR polymorphism (participation coefficient DMS), density plots and boxplots are presented for several proportional thresholds (5%, 10%, 15%, 20%, 25% and 30%). We observed that differences were only pronounced for lower proportional thresholds (0.01-0.06). Note the different axes. DMS, default mode subnetwork; prop. thres., proportional threshold.

Supplement 10

Figure 2: Density plots and boxplots for the main effect of the 5-HTTLPR polymorphism (participation coefficient of FPS)

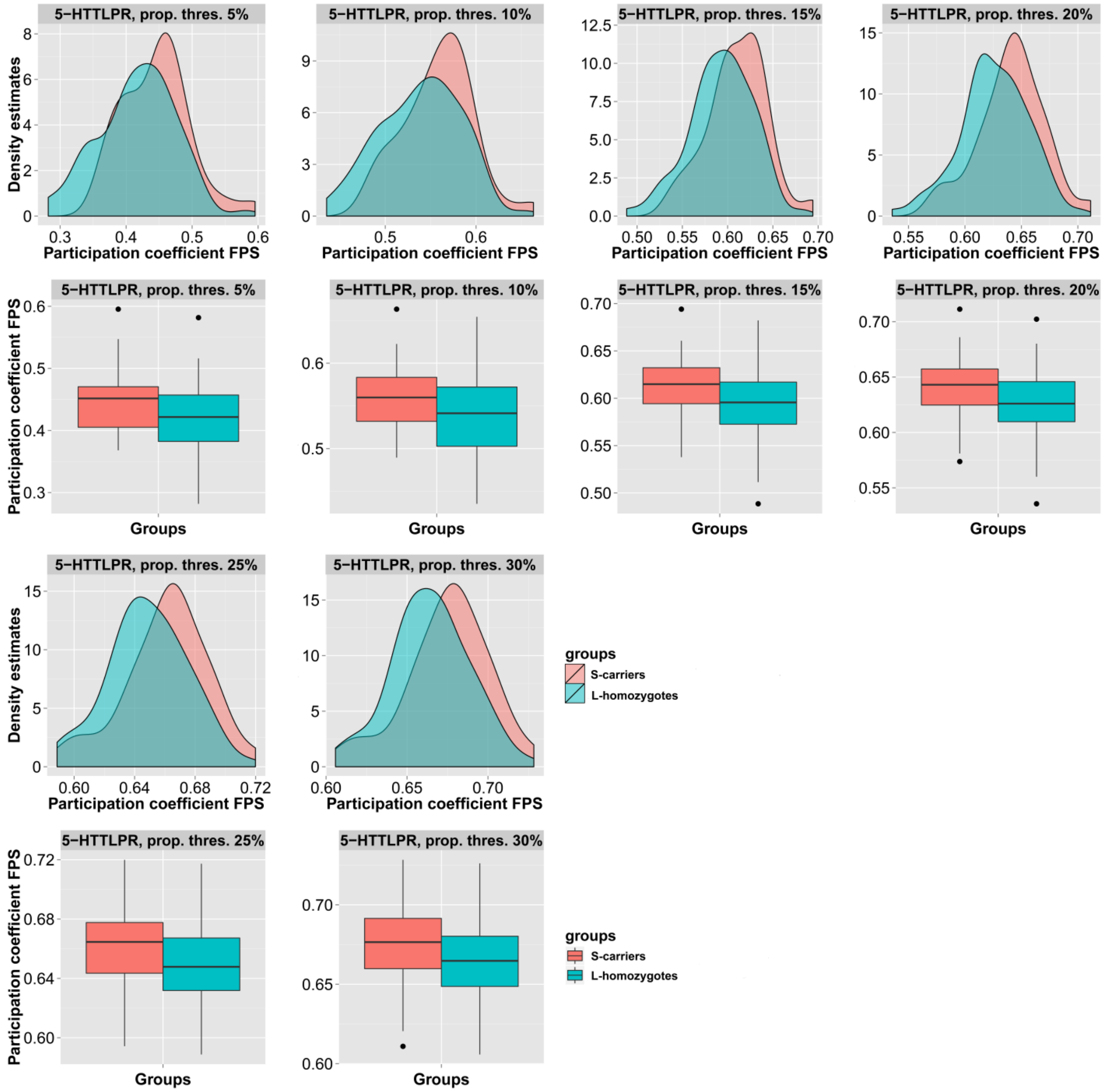

Figure 2 For the main effect of the 5-HTTLPR polymorphism (participation coefficient FPS), density plots and boxplots are presented for several proportional thresholds (5%, 10%, 15%, 20%, 25% and 30%). Note the different axes. FPS, frontal-parietal subnetwork; prop. thres., proportional threshold.

Supplement 11

Table 3: Correlation values for the association between the network measures and neuroticism per genetic group

	Proportional threshold in %																													
	**1**	**2**	**3**	**4**	**5**	**6**	**7**	**8**	**9**	**10**	**11**	**12**	**13**	**14**	**15**	**16**	**17**	**18**	**19**	**20**	**21**	**22**	**23**	**24**	**25**	**26**	**27**	**28**	**29**	**30**
**COMT Local efficiency, SMS**																														
Risk *r*	-.15	-.24	-.33	-.36	-.47	-.44	-.46	-.46	-.47	-.47	-.49	-.48	-.49	-.50	-.49	-.49	-.50	-.50	-.50	-.50	-.50	-.50	-.50	-.50	-.50	-.50	-.50	-.50	-.50	-.50
Non-risk *r*	-.11	-.13	-.05	-.01	-.01	-.04	-.13	-.12	-.11	-.13	-.15	-.15	-.14	-.14	-.12	-.13	-.13	-.13	-.13	-.14	-.14	-.14	-.15	-.15	-.15	-.14	-.15	-.15	-.15	-.15
**COMT Local efficiency, VS**																														
Risk *r*	-.31	-.29	-.28	-.32	-.39	-.43	-.44	-.44	-.45	-.46	-.46	-.46	-.45	-.47	-.47	-.47	-.47	-.47	-.47	-.47	-.47	-.47	-.47	-.47	-.47	-.47	-.47	-.47	-.47	-.47
Non-risk *r*	-.01	.01	.02	.04	.02	.00	-.01	-.02	-.03	-.03	-.03	-.04	-.05	-.05	-.07	-.08	-.08	-.09	-.09	-.09	-.09	-.09	-.09	-.10	-.10	-.10	-.11	-.11	-.11	-.11

Table 3 Correlation values for the association between the network measures and neuroticism per genetic group. Correlation values are given for each proportional threshold value. COMT, catechol-*O*-methyltransferase; SMS, somatosensory-motor subnetwork; VS, visual subnetwork.

Supplement 12

Figure 3: Bootstrap results for the interaction between the COMT polymorphism and neuroticism (local efficiency SMS)

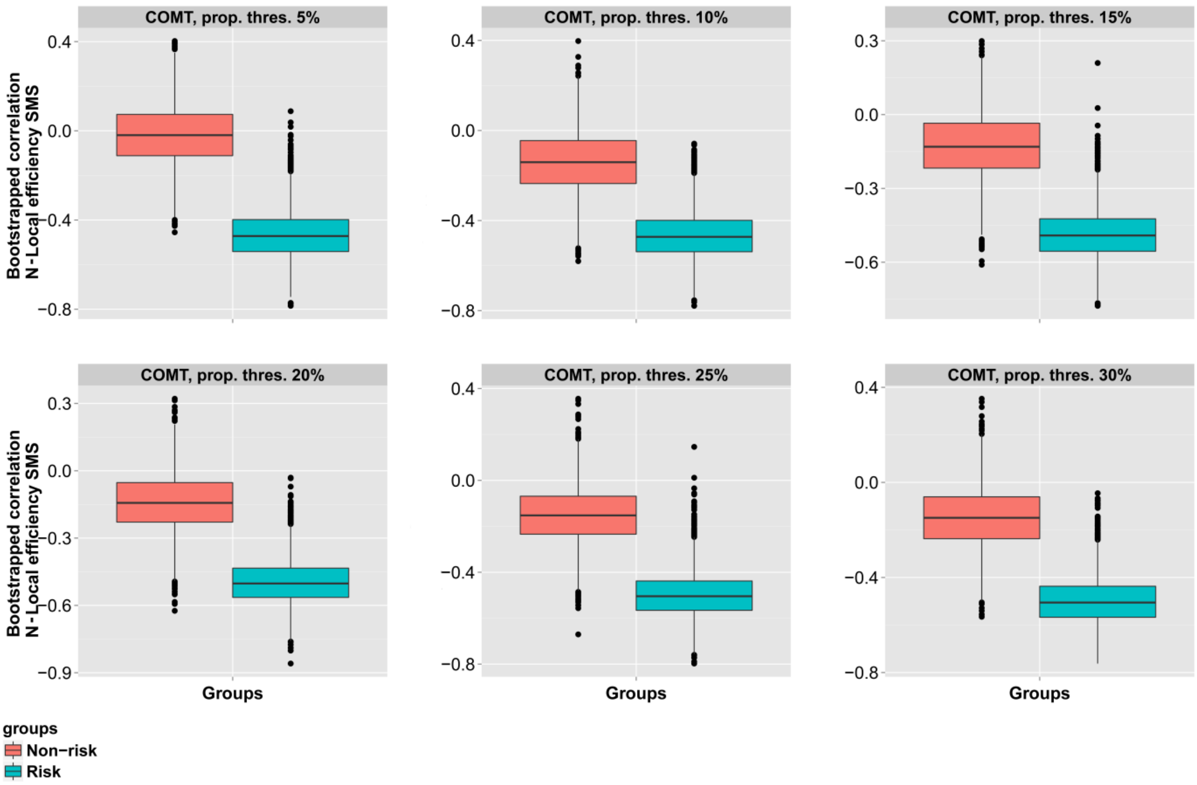

Figure 3 Bootstrapping (n=5000) was performed for the interaction between the COMT polymorphism and neuroticism for several proportional thresholds (5%, 10%, 15%, 20%, 25% and 30%). Note the different axes. N, neuroticism; prop. thres., proportional threshold; SMS, somatosensory-motor subnetwork.

Supplement 13

Figure 4: Bootstrap results for the interaction between the COMT polymorphism and neuroticism (local efficiency VS)

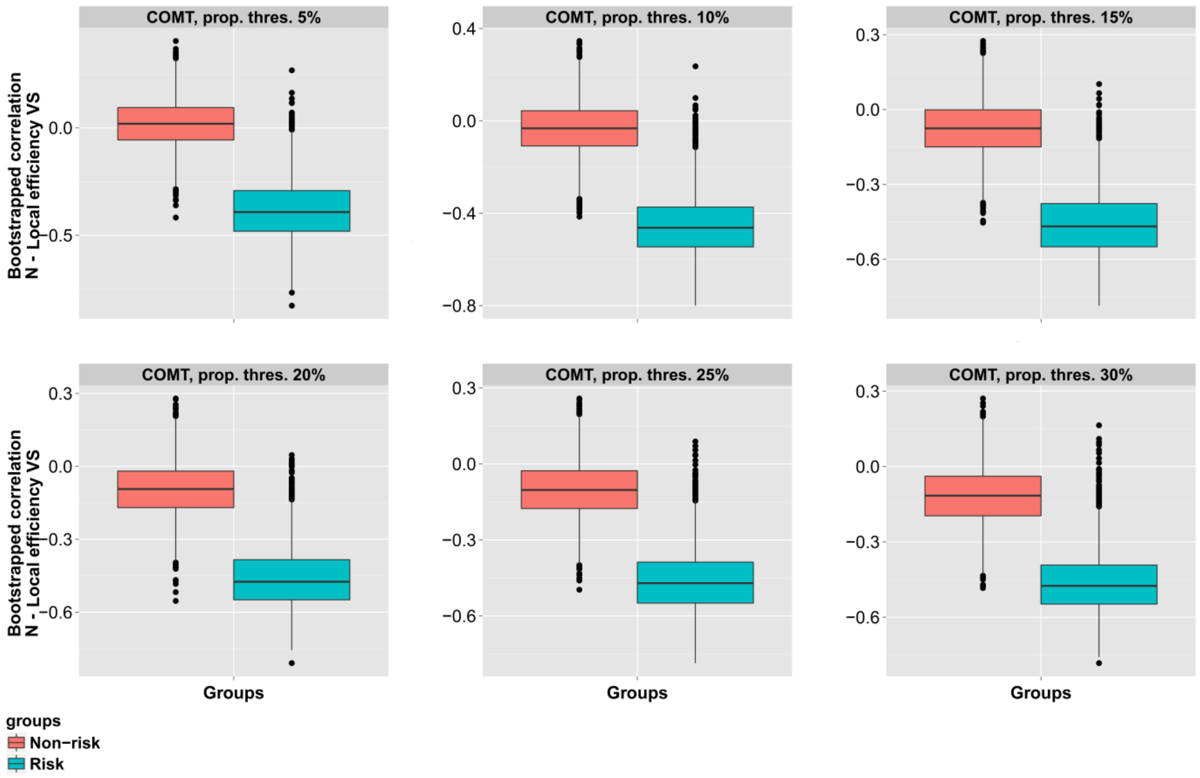

Figure 4 Bootstrapping (n=5000) was performed for the interaction between the COMT polymorphism and neuroticism for several proportional thresholds (5%, 10%, 15%, 20%, 25% and 30%). Note the different axes. N, neuroticism; prop. thres., proportional threshold; VS, visual subnetwork.

References

Ashburner, J. (2007). A fast diffeomorphic image registration algorithm. NeuroImage, 38(1), 95-113.

Blondel, V. D., Guillaume, J., Lambiotte, R., & Lefebvre, E. (2008). Fast unfolding of communities in large networks. Journal of Statistical Mechanics, Theory and Experiment, P10008.

Doornbos, B., Dijck-Brouwer, D. A., Kema, I. P., Tanke, M. A., van Goor, S. A., Muskiet, F. A., et al. (2009). The development of peripartum depressive symptoms is associated with gene polymorphisms of MAOA, 5-HTT and COMT. Progress in Neuro-Psychopharmacology & Biological Psychiatry, 33(7), 1250-1254.

Geerligs, L., Renken, R. J., Saliasi, E., Maurits, N. M., & Lorist, M. M. (2015). A brain-wide study of age-related changes in functional connectivity. Cerebral Cortex, 25(7), 1987-1999.

Hariri, A. R., Mattay, V. S., Tessitore, A., Kolachana, B., Fera, F., Goldman, D., et al. (2002). Serotonin transporter genetic variation and the response of the human amygdala. Science, 297(5580), 400-403.

Paulesu, E., Sambugaro, E., Torti, T., Danelli, L., Ferri, F., Scialfa, G., et al. (2010). Neural correlates of worry in generalized anxiety disorder and in normal controls: A functional MRI study. Psychological Medicine, 40(1), 117-124.

Pollatos, O., Herbert, B. M., Matthias, E., & Schandry, R. (2007). Heart rate response after emotional picture presentation is modulated by interoceptive awareness. International Journal of Psychophysiology, 63(1), 117-124.

Power, J. D., Barnes, K. A., Snyder, A. Z., Schlaggar, B. L., & Petersen, S. E. (2012). Spurious but systematic correlations in functional connectivity MRI networks arise from subject motion. NeuroImage, 59(3), 2142-2154.

Power, J. D., Cohen, A. L., Nelson, S. M., Wig, G. S., Barnes, K. A., Church, J. A., et al. (2011). Functional network organization of the human brain. Neuron, 72(4), 665-678.

Sanfey, A. G., Rilling, J. K., Aronson, J. A., Nystrom, L. E., & Cohen, J. D. (2003). The neural basis of economic decision-making in the ultimatum game. Science, 300(5626), 1755-1758.

Servaas, M. N., Geerligs, L., Renken, R. J., Marsman, J. B., Ormel, J., Riese, H., et al. (2015). Connectomics and neuroticism: An altered functional network organization. Neuropsychopharmacology, 40(2), 296-304.

Sun, Y., Danila, B., Josic, K., & Bassler, K. E. (2009). Improved community structure detection using a modified fine-tuning strategy. Europhysics Letters, 86(2), 28004.

Van Dijk, K. R., Hedden, T., Venkataraman, A., Evans, K. C., Lazar, S. W., & Buckner, R. L. (2010). Intrinsic functional connectivity as a tool for human connectomics: Theory, properties, and optimization. Journal of Neurophysiology, 103(1), 297-321.
